# Understanding Diagnostic Failure in Temporomandibular Disorders: A Narrative Synthesis of Evidence and Five‐Domain Framework for Iatrogenic Mismanagement

**DOI:** 10.1111/joor.70294

**Published:** 2026-08-26

**Authors:** Bernardo Correia Lima, Rayan Eboli Fortunato, Leonardo Brígido Metello Neves, Ricardo Grillo, Mariana Aparedida Brozoski, Fernando Melhem‐Elias, Janey Prodoehl, Louis G. Mercuri

**Affiliations:** ^1^ Department of Oral and Maxillofacial Surgery, Prosthesis and Traumatology University of São Paulo São Paulo Brazil; ^2^ Post Graduate Program, Department of Orofacial Pain Arthur Sá Earp Neto University Petrópolis Brazil; ^3^ Physical Therapy Program and College of Dental Medicine‐Illinois Midwestern University Downers Grove Illinois USA; ^4^ Department of Orthopaedic Surgery Rush University Medical Center Chicago Illinois USA

**Keywords:** clinical reasoning, dental education, diagnostic error, iatrogenic harm, orofacial pain, overtreatment, patient safety, temporomandibular disorders

## Abstract

**Background:**

Diagnostic errors in temporomandibular disorders (TMDs) contribute to unnecessary procedures and avoidable patient mismanagement despite advances in pain science, diagnostic classification systems, and evidence‐based care.

**Objective:**

To conduct a narrative review and present a dynamic, five‐domain framework explaining how diagnostic uncertainty interacts with educational, cognitive, systemic, and ethical factors to produce iatrogenic overtreatment.

**Methods:**

Searches of PubMed, Scopus, Web of Science, and Google Scholar (inception to April 2026) identified literature on TMD diagnosis, clinical reasoning, dental education, health system organization, and bioethics. Concepts were synthesized into interacting thematic domains.

**Results:**

Five dynamic, interacting domains were identified: (1) Educational Deficits, (2) Structural Reductionism, (3) Occlusal Overinterpretation, (4) System Fragmentation, and (5) Ethical Dissonance. The model demonstrates how educational gaps foster reductionist diagnostic paradigms, which are amplified by health‐system silos and defensive clinical practices, increasing the likelihood of non‐indicated, irreversible interventions.

**Conclusions:**

The Five‐Domain Framework offers a systemic model for understanding diagnostic breakdown in TMD management, providing a structured foundation for educational reform, integrated care pathways, and future empirical research.

## Graphical abstract

Schematic flowchart illustrating the Five‐Domain Framework for understanding diagnostic failure and iatrogenic overtreatment in temporomandibular disorders (TMDs). Upstream determinants (Educational Deficits, System Fragmentation) create a baseline of diagnostic uncertainty when encountering non‐specific TMD presentations. Cognitive heuristics, specifically Structural Reductionism (overreliance on imaging) and Occlusal Overinterpretation (attributing pain to bite anomalies), act as reinforcing biases that convert uncertainty into diagnostic error. Amplified by external drivers (financial incentives, defensive practice, and patient anxiety), this results in an overtreatment cascade of non‐indicated, irreversible interventions. The progression culminates in ethical dissonance (compromised informed consent and non‐maleficence) and terminal iatrogenic harm, creating a financial, physical, and psychological burden for the patient.

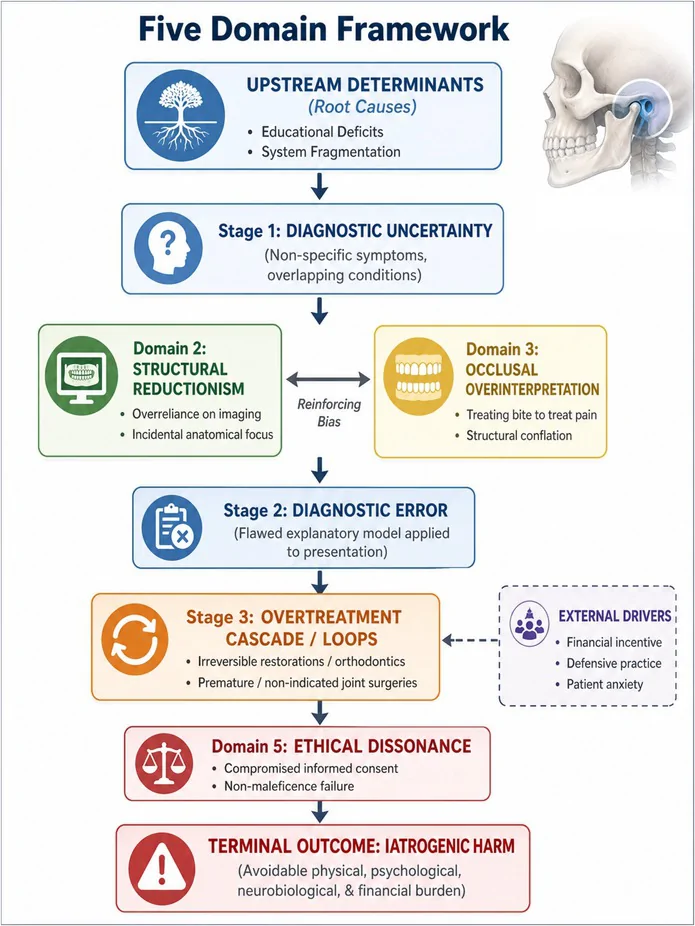

## 1 Introduction

Temporomandibular disorders (TMDs) represent a cluster of musculoskeletal and neuromuscular conditions involving the temporomandibular joints (TMJs), masticatory muscles, and associated structures. Characterized by high prevalence and clinical complexity, TMDs are recognized as a leading cause of non‐dental orofacial pain globally [1, 2]. Epidemiological data, including findings from the multi‐centre Orofacial Pain Prospective Evaluation and Risk Assessment (OPPERA) study, highlight that TMD presentation is frequently multifactorial, overlapping with systemic pain conditions, psychological distress, and sleep disturbances [3]. Standardized diagnostic systems, notably the Diagnostic Criteria for Temporomandibular Disorders (DC/TMD) [3] and the International Classification of Orofacial Pain [1], have established highly reliable, validated criteria that distinguish localized joint pathologies from myofascial and neurogenic pain.

Despite the availability of these rigorous diagnostic instruments, diagnostic variability and uncertainty remain widely recognized challenges across diverse dental and medical settings. The landmark report of the National Academies of Sciences, Engineering, and Medicine, Temporomandibular Disorders: Priorities for Research and Care [2], emphasized that individuals with TMDs face significant barriers to receiving accurate, timely diagnoses. Because TMD symptoms often mimic odontogenic diseases or present as non‐specific craniofacial pain, clinicians frequently encounter diagnostic uncertainty. This uncertainty is exacerbated by the lack of universally adopted, mandatory predoctoral competencies in orofacial pain, leaving many practitioners ill‐equipped to interpret complex pain phenotypes.

When diagnostic uncertainty is managed through historical conceptual models, the risk of overtreatment increases substantially. Decades of clinical literature, spearheaded by foundational scholars such as Charles S. Greene and Daniele Manfredini, have documented a historical tendency within dental specialties to address orofacial pain through irreversible structural or occlusal changes. As noted by Greene and Manfredini [4], the persistence of non‐evidence‐based therapies, such as extensive full‐mouth restorative rehabilitations, prolonged orthodontic movements, or premature or non‐indicated surgical interventions under diagnostic uncertainty, frequently stems from an initial failure to correctly differentiate primary orofacial pain from localized anatomical variations.

While overtreatment and its associated financial and physical burdens have been extensively discussed, less attention has been directed toward understanding the precise structural and cognitive mechanisms that transform initial diagnostic uncertainty into irreversible interventions. The purpose of this article is therefore to synthesize existing clinical, educational, and healthcare‐system literature and propose a conceptual framework explaining these mechanisms. By moving beyond criticisms of specific treatment modalities, this paper delineates the upstream pathways that shape diagnostic behaviour, offering a systematic paradigm to mitigate iatrogenic harm.

## 2 Methodological Approach

This article is a narrative review with conceptual synthesis; it is not a systematic or scoping review, and no formal quality appraisal or quantitative pooling was undertaken. The approach is reported transparently to allow readers to judge its scope and limitations.

### 2.1 Search Strategy and Selection Criteria

Comprehensive electronic searches were conducted in PubMed, Scopus, Web of Science, and Google Scholar from database inception to April 2026. The search strategy utilized combinations of MeSH terms and free‐text keywords: (“temporomandibular joint disorders” OR “orofacial pain”) AND (“diagnostic error” OR “overtreatment” OR “clinical reasoning” OR “dental education” OR “dental occlusion” OR “referral pathways” OR “patient safety” OR “bioethics”).

The search yielded approximately 420 records. Following title, abstract, and full‐text screening, 86 primary studies, systematic reviews, authoritative consensus reports (e.g., DC/TMD, ICOP, NASEM), and bioethical analyses were included. Literature was selected based on its explicit focus on TMD diagnostic reasoning, educational competencies, healthcare delivery structures, or ethical dimensions of interventionism.

### 2.2 Conceptual Synthesis and Conflict Analysis

Concepts were extracted and synthesized through iterative thematic analysis. Literature offering alternative or conflicting viewpoints, such as studies supporting early mechanical stabilization, historical occlusal theories, or early surgical intervention in internal derangement, was explicitly evaluated to prevent confirmation bias. The resulting framework categorizes explanatory drivers across five primary domains, distributed across the included core literature as follows:

- **Educational Deficits:** AAOP Committee et al. [5], Costa et al. [6], Gnauck et al. [7], NASEM [2], Tormes et al. [8]
- **Structural Reductionism:** Dolwick et al. [9], Ilgunas et al. [10], Lau et al. [11], Makdissi et al. [12], Mercuri [13]
- **Occlusal Overinterpretation:** Greene and Manfredini [4], Manfredini et al. [14], McNamara et al. [15], Pascu et al. [16]
- **System Fragmentation:** Correia Lima et al. [17], Manfredini et al. [18], NASEM [2], Patel et al. [19]
- **Ethical Dissonance:** Greene and Manfredini [20], Mercuri [13], Reid and Greene [21], Renton et al. [22]

## 3 Evolution of Diagnostic Thinking in TMD

The conceptualization of TMD has undergone a substantial paradigm shift over the past half‐century. Understanding this evolution is essential to understanding why outdated models continue to influence contemporary practice.

### 3.1 The Historical Era: The Occlusal and Mechanical Paradigms

For much of the 20th century, TMD was viewed primarily through a mechanical lens [15]. Originating from early concepts of “Costen's syndrome,” symptoms were attributed to alterations in dental occlusion, maxillomandibular relationships, and mechanical disc displacement. Within this paradigm, pain was conceptualized as a direct consequence of structural misalignment. Consequently, treatment focused primarily on restoring a theoretical “ideal” occlusion or surgically correcting internal joint derangements. This era facilitated the clinical acceptance of invasive, irreversible dental interventions as standard first‐line therapies.

### 3.2 The Transitional Era: Introduction of Biopsychosocial Standardized Criteria

The publication of the Research Diagnostic Criteria for Temporomandibular Disorders (RDC/TMD) in 1992 marked a pivotal shift away from pure reductionism. The RDC/TMD introduced a dual‐axis diagnostic system: Axis I focused on physical clinical findings, while Axis II evaluated psychological status and pain‐related disability. This dual‐axis model formally introduced the biopsychosocial model to dental medicine, recognizing that psychosocial factors could significantly modulate pain perception and chronification independently of peripheral anatomical findings [3].

### 3.3 The Modern Era: Validated Multi‐Dimensional Frameworks

Contemporary orofacial pain medicine operates under highly refined, validated frameworks. The DC/TMD, expanded from the RDC/TMD, provides evidence‐based protocols for clinical utility, demonstrating high sensitivity and specificity for the most common TMD subtypes [3]. Concurrently, the ICOP [1] and data from the longitudinal OPPERA study have further contextualized TMD within the wider spectrum of central pain processing, highlighting genetic, neurobiological, and environmental vulnerabilities. Pain is now understood not merely as a reflection of tissue damage, but often as a manifestation of central sensitization and somatosensory dysfunction.

The persistence of older explanatory models alongside these contemporary frameworks suggests that scientific advances alone are insufficient to alter clinical behaviour. Despite consensus guidelines emphasizing conservative, reversible management, mechanical and occlusal paradigms remain prevalent in clinical settings, establishing a clear need for an explanatory framework regarding diagnostic inertia (Table 1).

**TABLE 1:** Evolution of TMD diagnostic models.

Era	Dominant model	Strengths	Limitations
Historical Era	Occlusal and mechanical paradigm [15]	Provided clear, actionable mechanical treatment protocols; established dental ownership over TMD care	Lacked scientific validation; ignored neurobiological and psychosocial dimensions; led to significant iatrogenic harm
Transitional Era	Dual‐axis framework (RDC/TMD, 1992) [23]	Formally introduced the biopsychosocial model; added Axis II to quantify psychological comorbidity and pain disability	Primarily limited to research settings; insufficient sensitivity/specificity for complex or systemic clinical presentations
Modern Era	Validated multi‐dimensional frameworks: DC/TMD, ICOP, OPPERA [1, 3]	High sensitivity/specificity; clear diagnostic algorithms; integrates central sensitization, genetics, and systemic comorbidity	High implementation inertia; inconsistent adoption across predoctoral dental education and general clinical practice

## 4 Why Diagnostic Failure Persists

To understand why diagnostic failure and subsequent iatrogenic interventions persist despite robust scientific evidence, it is necessary to examine the cognitive, sociological, and institutional factors that shape clinical decision‐making.

### 4.1 Cognitive Biases

Clinicians operating under high clinical uncertainty frequently rely on cognitive heuristics. Confirmation bias leads practitioners to interpret non‐specific anatomical features (e.g., minor occlusal variations or asymptomatic disc displacement on MRI) as the definitive cause of a patient's pain. Anchoring bias causes clinicians to focus preferentially on the initial complaint (e.g., “my bite feels off”) without exploring systemic or neurobiological contributors [10].

### 4.2 Professional Identity

Historically, dental education and practice have prioritized procedural and technical interventions. This procedural identity can lead to an interventionist approach, in which therapeutic success is equated with physical action or structural alteration rather than diagnostic observation and conservative tracking [21].

### 4.3 Educational Inertia

Predoctoral dental curricula remain largely centred on traditional mechanical disciplines, with inconsistent integration of pain neuroscience and modern evidence‐based biopsychosocial diagnostic frameworks [6].

### 4.4 Referral Barriers and Healthcare Fragmentation

Clear, coordinated pathways between general dentistry, dental specialties, and allied healthcare disciplines (such as neurology, physical therapy, and clinical psychology) are frequently inadequate [17, 19]. These systemic gaps isolate clinicians within separate professional tracks, encouraging localized, single‐specialty approaches to complex, multi‐system pain conditions.

## 5 The Five‐Domain Framework

Rather than a linear sequence, the Five‐Domain Framework operates as a dynamic, interacting system with circular feedback loops. Upstream factors (Educational Deficits, System Fragmentation) interact with cognitive heuristics (Structural Reductionism, Occlusal Overinterpretation) and external drivers, including patient expectations [18], financial incentives, and defensive medico‐legal practices, to reinforce diagnostic errors and ethical misalignment (Table 2).

**TABLE 2:** The five‐domain framework.

Domain	Operational definition	Primary mechanism	Clinical manifestation	Preventive and mitigation strategy
1. Educational Deficits	Insufficient integration of pain neuroscience and biopsychosocial frameworks in predoctoral dental curricula [5, 6]	Relying on historical, mechanical paradigms due to lack of training in non‐odontogenic pain [7]	Inability to differentiate myofascial/neuropathic pain from dental or joint pathology [8]	Implementation of mandatory predoctoral orofacial pain core competencies [2, 5]
2. Structural Reductionism	Attributing complex pain phenotypes solely to localized anatomical variations [10]	Misinterpreting incidental or non‐specific imaging findings as definitive pain sources [11, 12]	Execution of structural/surgical interventions for asymptomatic anatomical features [9, 13]	Restricting imaging interpretations to clear indications (e.g., trauma, arthritis, neoplasia); correlating findings strictly with DC/TMD Axis I criteria [3, 13]
3. Occlusal Overinterpretation	Overestimating the causal role of dental bite relationships in TMD pain aetiology [14]	Conflating static/dynamic bite variations with active chronic pain pathology [4]	Irreversible occlusal adjustment, prosthodontics, or orthodontics for primary pain management [15, 16]	Promoting reversible management; adopting clinical guidelines decoupling routine TMD care from irreversible bite modification [4]
4. System Fragmentation	Lack of structured interdisciplinary coordination and referral pathways across specialties [19]	Isolation within specialty silos, creating disjointed clinical handoffs and delays [17]	Multiple conflicting opinions, serial procedures, and protracted care pathways [2, 18]	Establishing centralized interdisciplinary triage networks connecting general dentistry, OMFS, PT, and pain medicine [2, 17]
5. Ethical Dissonance	Compromising professional obligations (non‐maleficence, autonomy) under diagnostic uncertainty [20]	Prioritizing procedural action over diagnostic observation; incomplete communication of uncertainty [21]	Inappropriate procedural delivery; incomplete informed consent lacking non‐invasive option disclosure [13, 22]	Formal ethics training in managing diagnostic uncertainty; enforcing shared decision‐making models [20, 21]

### 5.1 Domain 1: Educational Deficits

Educational deficits serve as a fundamental upstream driver within this model. Predoctoral dental education historically lacks systematic integration of modern pain neuroscience and biopsychosocial frameworks [5, 6]. Students frequently receive fragmented instruction across independent clinical departments, leaving them reliant on historical mechanical paradigms. Empirical evaluations confirm that general practitioners and predoctoral students regularly demonstrate limited knowledge and diagnostic self‐efficacy regarding non‐odontogenic orofacial pain [7, 8]. Educational gaps create a baseline vulnerability wherein clinicians struggle to identify complex, non‐odontogenic pain phenotypes [2].

### 5.2 Domain 2: Structural Reductionism

Structural reductionism describes the cognitive inclination to attribute complex pain phenotypes exclusively to localized anatomical or mechanical abnormalities [10]. Clinically, this often manifests as an over‐reliance on advanced imaging modalities, such as magnetic resonance imaging (MRI) or cone‐beam computed tomography (CBCT).

It is critical to emphasize that imaging is essential and clinically indispensable in selected presentations, including suspected inflammatory arthritides, trauma, neoplasia, progressive degenerative joint disease, ankylosis, persistent structural lock, or surgical planning [13].

The clinical error lies not in the utility of imaging itself, but in the overinterpretation of incidental or non‐specific findings (such as asymptomatic disc displacement or minor osteophytic flattening) in patients whose pain phenotype is driven primarily by central sensitization or myofascial mechanisms [11, 12]. When non‐specific structural variants are treated as primary pain generators, invasive procedures may be performed without addressing the true underlying aetiology [9, 13].

### 5.3 Domain 3: Occlusal Overinterpretation

This domain addresses the historical conflation between dental occlusion and TMD aetiology. Contemporary evidence requires a nuanced differentiation across four distinct clinical contexts:

1. **Occlusion as a Population Risk Factor:** Large‐scale epidemiological and prospective cohort studies indicate that static or dynamic occlusal features represent weak, low‐level risk factors for the onset of TMD at the population level [14].
2. **Occlusal Function and Adaptation:** Occlusal factors play an important role in masticatory function and structural adaptation in individual patients, particularly during complex restorative or orthodontic rehabilitation [15].
3. **Irreversible Occlusal Therapy:** Current evidence does not support irreversible occlusal alterations (e.g., occlusal equilibration, full‐mouth reconstruction, or orthodontic retreatment) as first‐line or primary treatments for TMD pain [4, 15, 16].
4. **Reversible Appliances:** Reversible modalities, such as splints and stabilization appliances, maintain a legitimate therapeutic role for symptom relief, burden reduction, or joint unloading, provided they are not interpreted as diagnostic “proof” of an underlying occlusal pathology.

### 5.4 Domain 4: System Fragmentation

System fragmentation captures structural deficiencies within healthcare delivery, characterized by isolated specialty silos and lack of coordinated interdisciplinary care [2, 17, 19]. General dentists, orthodontists, prosthodontists, oral and maxillofacial surgeons, neurologists, physical therapists, and pain specialists frequently practice without standardized cross‐disciplinary triage protocols. Survey and referral analyses show that patients navigating fragmented care experience diagnostic delays, contradictory specialist opinions, and repeated low‐value interventions [17, 18]. Notably, patients initially consulting clinicians focused on structural or bite adjustments visit significantly more providers before obtaining an accurate diagnosis [18].

### 5.5 Domain 5: Ethical Dissonance

Ethical Dissonance is defined as the operational misalignment between professional ethical duties and actual clinical decision‐making when diagnostic uncertainty is managed through procedural intervention [20, 21]. To ensure conceptual clarity, Ethical Dissonance must be distinguished from related constructs:

- **Malpractice:** Refers to legal negligence or failure to adhere to standard care parameters resulting in direct injury.
- **Overtreatment/Low‐Value Care:** Refers to the quantitative delivery of healthcare services that provide minimal net benefit relative to cost or risk.
- **Ethical Dissonance:** Uniquely describes the internal and systemic compromise of moral agency. It occurs when a clinician, faced with diagnostic ambiguity or patient distress, relies on procedural intervention to provide immediate action, rationalizing non‐evidence‐based therapies through outdated models [20].

In this context, informed consent must be conceptualized not merely as a formal disclosure of procedural risks, but as the active, transparent communication of diagnostic uncertainty, self‐limiting disease trajectories, and conservative, non‐invasive alternatives [21]. Across specialties, ethical dissonance manifests in iatrogenic loops: endodontic procedures executed on sound teeth for referred muscle pain [22], repeated orthodontic retreatments for bite stabilization, or non‐indicated open‐joint surgeries under diagnostic uncertainty [13].

## 6 Relationship to Existing Models

The Five‐Domain Framework builds directly upon foundational analyses of overtreatment in orofacial pain [4, 20, 21]. Prior work established that overtreatment in TMD care is not merely an isolated technical error, but a systemic issue rooted in historical paradigms [4].

The present framework contributes three distinct conceptual advances:

1. It explicitly connects upstream educational deficits [5, 6] and healthcare delivery structures [17, 19] directly to downstream clinical decision‐making.
2. It details the cognitive mechanisms, specifically structural reductionism [10, 11] and occlusal overinterpretation [14, 16], through which diagnostic error occurs.
3. It integrates ethical dissonance [20, 21] as an observable outcome of diagnostic breakdown rather than a separate bioethical commentary, providing a comprehensive systems model.

By establishing this explicit, ordered link between systemic factors, individual cognitive errors, and ethical dissonance, the model maps the pre‐procedural pathway and articulates how and why clinical reasoning breaks down (Table 3).

**TABLE 3:** Representative literature supporting each domain.

Domain	Key publications	Main contribution to the framework
1. Educational Deficits	AAOP Committee [5]; Costa et al. [6]; Gnauck et al. [7]; NASEM [2]; Tormes et al. [8]	Document the absence of standardized competencies and provide empirical evidence of poor diagnostic performance among inadequately trained practitioners
2. Structural Reductionism	Dolwick et al. [9]; Ilgunas et al. [10]; Lau et al. [11]; Makdissi et al. [12]; Mercuri [13]	Show that incidental imaging findings fail to match clinical symptom maps, warning against structural targets in pain treatment
3. Occlusal Overinterpretation	Greene and Manfredini [4]; Manfredini et al. [14]; McNamara et al. [15]; Pascu et al. [16]	Provide systematic and scoping syntheses confirming that occlusion does not predict or cause most contemporary TMD presentations
4. System Fragmentation	Correia Lima et al. [17]; Manfredini et al. [18]; NASEM [2]; Patel et al. [19]	Identify multi‐provider pathway flaws and structural barriers that transform diagnostic gaps into prolonged referral cascades
5. Ethical Dissonance	Greene and Manfredini [20]; Mercuri [13]; Reid and Greene [21]; Renton et al. [22]	Detail localized, multi‐specialty overtreatment patterns (surgical, orthodontic, endodontic) and frame their execution under diagnostic uncertainty as an ethical dissonance

## 7 Implications for Clinical Practice and Education

### 7.1 Clinical Implications

Faced with diagnostic uncertainty, the framework suggests that clinicians should default to conservative, fully reversible management strategies. Applying the diagnostic criteria of the DC/TMD can protect patients from premature structural interventions while diagnostic clarity is pursued [3].

### 7.2 Educational Implications

Dental curricula could benefit from adopting a standardized, interdisciplinary approach to orofacial pain [5]. Shifting competencies from mechanical adjustments toward pain neuroscience and biopsychosocial assessment could meaningfully reduce the prevalence of reductionist thinking in future practitioners [6].

### 7.3 Systems Implications

Healthcare institutions and professional organizations may reduce iatrogenic risk by implementing structured multidisciplinary referral pathways [2, 17]. Integrating dental and medical triage systems would help ensure that patients with complex, overlapping pain phenotypes bypass uncoordinated specialty tracks and access comprehensive care early [19].

## 8 Future Research Agenda

To advance from a conceptual model to empirical application, three primary research pathways are proposed:

1. **Validation study**. Empirical research is required to evaluate whether independent clinicians can reliably classify complex patient cases and histories of overtreatment according to the domains of this framework, establishing its reliability and clinical utility.
2. **Educational study**. Longitudinal studies could evaluate whether the formal integration of multi‐dimensional pain curricula reduces the prevalence of structural reductionism and occlusal overinterpretation among dental graduates.
3. **Health‐system study**. Comparative health‐services research could assess whether the implementation of integrated, multidisciplinary triage and referral pathways significantly reduces the incidence of iatrogenic interventions and lowers overall costs compared with traditional fragmented care models.

## 9 Limitations

While this framework offers a structured approach to analysing diagnostic breakdown, several limitations must be acknowledged.

- **Interpretive and conceptual nature**. The framework is fundamentally interpretive, serving as a conceptual model rather than a direct reflection of quantified causal relationships.
- **Absence of causal testing**. The sequential relationships proposed between the domains (e.g., the cascade from educational deficits to cognitive error) have not been empirically tested or quantified.
- **Narrative methodology**. As a narrative review, this synthesis did not use a pre‐registered protocol, formal risk‐of‐bias appraisal, or systematic screening, and is therefore subject to selection and interpretation bias; it relies in part on qualitative, opinion‐based, and consensus literature that may reflect the paradigm preferences of leading experts.
- **Requirement for empirical validation**. The model serves as a starting hypothesis and requires rigorous, prospective empirical validation across diverse clinical and educational settings before its widespread adoption can be fully recommended.

## 10 Conclusions

Diagnostic failures in TMD care appear to arise from interacting educational, cognitive, clinical, and healthcare‐system factors. The Five‐Domain Framework proposed in this article offers a structured explanation of how these factors may converge to produce overtreatment and iatrogenic harm. By shifting attention from individual clinical decisions to the broader mechanisms that shape diagnostic behaviour, the framework provides a foundation for future educational, clinical, and research initiatives aimed at improving patient care.

## Author Contributions

**Janey Prodoehl:** writing – review and editing, visualization, data curation. **Louis G. Mercuri:** writing – review and editing, writing – original draft, supervision, data curation, visualization. **Rayan Eboli Fortunato:** conceptualization, methodology, formal analysis. **Leonardo Brígido Metello Neves:** methodology, writing – original draft, formal analysis. **Bernardo Correia Lima:** conceptualization, investigation, writing – review and editing, writing – original draft, methodology, formal analysis. **Ricardo Grillo:** writing – review and editing, visualization. **Fernando Melhem‐Elias:** project administration. **Mariana Aparedida Brozoski:** writing – review and editing, visualization.

## Funding

The authors have nothing to report.

## Conflicts of Interest

The authors declare no conflicts of interest. Dr. Mercuri is a Clinical Consultant for Stryker/TMJ Concepts (Ventura, CA).

## Data Availability

Data sharing is not applicable to this article as no new datasets were generated or analysed during this study.
